# Effect of Gamma Ray Irradiation on Friction Property of Poly(vinyl alcohol) Cast-Drying on Freeze-Thawed Hybrid Gel

**DOI:** 10.3390/gels4020030

**Published:** 2018-03-29

**Authors:** Saori Sasaki, Seiji Omata, Teruo Murakami, Naotsugu Nagasawa, Mitsumasa Taguchi, Atsushi Suzuki

**Affiliations:** 1 Research Center for Advanced Biomechanics, Kyushu University, 744 Motooka, Nishi-ku, Fukuoka 819-0395, Japan; s-omata@mech.nagoya-u.ac.jp (S.O.); tmura@mech.kyushu-u.ac.jp (T.M.); 2 Institute for Material Chemistry and Engineering, Kyushu University, 744 Motooka, Nishi-ku, Fukuoka 819-0395, Japan; 3 Department of Micro-Nano Mechanical Science and Engineering, Nagoya University, Furo-cho, Chikusa-ku, Nagoya 464-8603, Japan; 4 Faculty of Fukuoka Medical Technology, Teikyo University, 6-22 Misaki-machi, Omuta 836-8505, Japan; 5 Takasaki Advanced Radiation Research Institute, National Institutes for Quantum and Radiological Science and Technology, Watanuki 1233, Takasaki, Gunma 370-1292, Japan; nagasawa.naotsugu@qst.go.jp (N.N.); taguchi.mitsumasa@qst.go.jp (M.T.); 6 Research Institute of Environment and Information Sciences, Yokohama National University, 79-7 Tokiwadai, Hodogaya-ku, Yokohama 240-8501, Japan; asuzuki@ynu.ac.jp

**Keywords:** PVA gel, gamma ray sterilization, artificial hydrogel cartilage, frictional property, wear

## Abstract

Poly(vinyl alcohol) (PVA) is a biocompatible polymer with low toxicity. It is possible to prepare physically cross-linked PVA gels having hydrogen bonds without using a cross-linking agent. The newly reported physically cross-linked PVA cast-drying (CD) on freeze-thawed (FT) hybrid gel has an excellent friction property, which is expected to be applied as a candidate material for artificial cartilage. Gamma ray sterilization for clinical applications usually causes additional chemical cross-linking and changes physical properties of gels. In this study, CD on FT hybrid gels were irradiated using gamma rays at a different dose rate and irradiance. The results showed the optimized irradiation conditions for gamma irradiated gels to retain excellent friction characteristics.

## 1. Introduction

Poly(vinyl alcohol) (PVA) is a high biocompatible synthetic polymer with low toxicity and excellent mechanical properties. Physically cross-linked PVA gels having hydrogen bonds are prepared without using a cross-linking agent via two methods. One is the repeated freeze-thawing (FT) of aqueous PVA solutions [1], while the other one is the cast-drying (CD) of them [2,3]. These two physically cross-linked PVA gels have three-dimensional amorphous network structures which are physically cross-linked by microcrystallites. The distributions of microcrystallites, however, are quite different. While FT gel has a heterogeneous network structure with high permeability, CD gel has a uniform network structure with low permeability [4]. The possibility for application of FT gels is already reported as candidate materials for artificial cartilage, such as hip and knee prostheses [5,6,7]. It is found that FT gels show a good frictional property similar to the natural articular cartilage. On the other hand, CD gels reduce the friction level compared with FT gels and the natural articular cartilage in reciprocating friction tests in saline solution [8,9]. The coefficient of friction of CD gel in saline solution was about 0.05, which was superior to FT gel (0.20) and natural articular cartilage (0.15) [9].

Recently, a method for preparing the physically cross-linked PVA gel as PVA CD on FT hybrid gel was newly reported by Suzuki et al. [10]. The CD gel was laminated on the FT gel. This PVA CD on FT hybrid gel presents excellent friction properties in reciprocating friction tests in water and physiological saline [4,10,11]. The coefficient of friction of PVA CD on FT hybrid gel was in a lower range than 0.005 and the wear was minimal in the simulated synovial fluid, whereas that of FT and CD gel reached 0.012 and 0.01, respectively [12]. Therefore, such PVA CD on FT hybrid gel is expected to be applied as a candidate material for artificial cartilage.

In clinical applications, sterilization treatment is a mandatory procedure. There are several methods for sterilization. Autoclave sterilization is a widely used one in medical fields. The microorganisms are killed by heating with saturated steam at an appropriate temperature over 121 °C in a sealed apparatus. The advantage of this method is that heat can penetrate quickly into the deep part of the object and surely kill microorganisms in a short time. Therefore, this method is widely used in medical institutions and pharmaceutical manufacturing sites. However, heating using saturated steam destroys the microcrystallines as cross-linked regions. Therefore, autoclave sterilization cannot be used for physically cross-linked PVA gels. Besides, in a previous study, we soaked a hybrid gel into 70% ethanol solution for sterilization for animal experiments. The friction property of the CD on FT hybrid gel, however, was deteriorated due to shrinkage of gel surface [13]. Therefore, gamma ray sterilization is considered as a suitable method. There are many reports on gamma ray sterilization of gels [14,15]. Moderate gamma ray sterilization does not destroy microcrystals, and the gel shape is possible to maintain. Nevertheless, additional chemical cross-linking could occur, so the physical properties of PVA CD on FT hybrid gels are expected to change after gamma ray irradiation [16,17,18]. Therefore, it is necessary to investigate the effect of gamma rays on frictional property of PVA CD on FT hybrid gels.

In this study, we irradiated the PVA CD on FT hybrid gels under different conditions, including varied irradiation rate and total irradiance, to investigate the relationship between gamma ray irradiation and frictional properties. Then, friction and wear tests were performed in a reciprocating friction test at two lubricant temperatures, i.e., room temperature and body temperature (37 °C).

## 2. Results and Discussion

We irradiated the gels using gamma rays under various conditions of irradiation rate and total irradiance as described in Section 4.1.2 (Gamma Ray-Irradiation). In order to evaluate the tribological properties for artificial cartilage, frictional properties were characterized by the reciprocating friction test in this study. In order to evaluate the wear and durability, the sample surface was observed using a phase contrast microscope after completion of the reciprocating friction test. From the obtained results, we investigated the changes of friction and wear characteristics of the gels irradiated using gamma rays and achieved the optimum irradiation conditions.

### 2.1. Swelling Property

**Figure 1:**
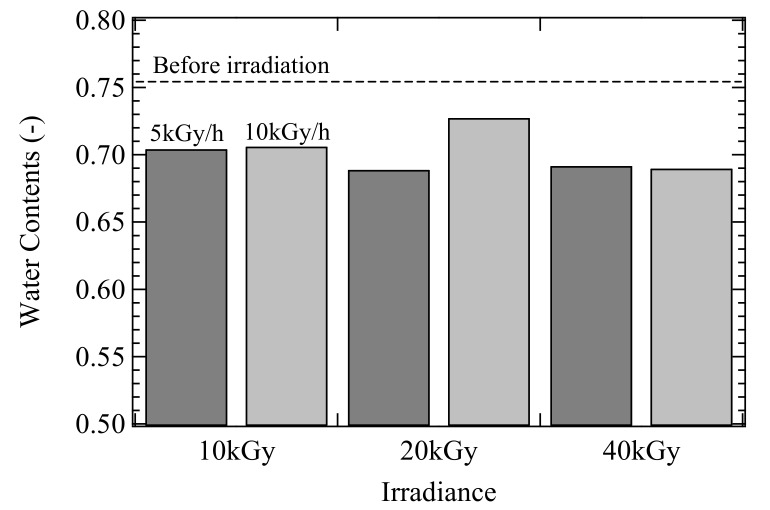
Water contents of poly(vinyl alcohol) PVA cast-drying (CD) on freeze-thawed (FT) hybrid gels before and after gamma ray irradiation. The broken line shows the water content of the gel before gamma irradiation.

One of the characteristics of PVA gels is a water content as high as about 70–80%, which is close to that of articular cartilage in human body (about 70–80%) [19]. The change of water content before and after gamma ray irradiation was investigated. The result is shown in Figure 1. Before gamma ray irradiation, the water content of PVA CD on FT hybrid gels reached 75%. All samples after gamma ray irradiation showed lower water contents than the ones before irradiation. The reduction in the water contents was caused by the process in which additional chemically cross-linking was introduced during gamma ray irradiation. The water content tended to decrease slightly as the irradiance of gamma rays increased.

Although the water content decrement occurred after irradiation using gamma rays, it was maintained around 70%, which is close to that of articular cartilage. Therefore, the PVA CD on FT hybrid gel after gamma ray irradiation can be potentially used for artificial cartilage with biphasic property. It is considered that the layered structure composing of CD and FT gel layers with appropriate microcrystallites maintained the necessary water content. Further investigations to elucidate the swelling behaviors after gamma ray irradiation are required.

### 2.2. Frictional Properties

#### 2.2.1. Reciprocating Test at Room Temperature

**Figure 2:**
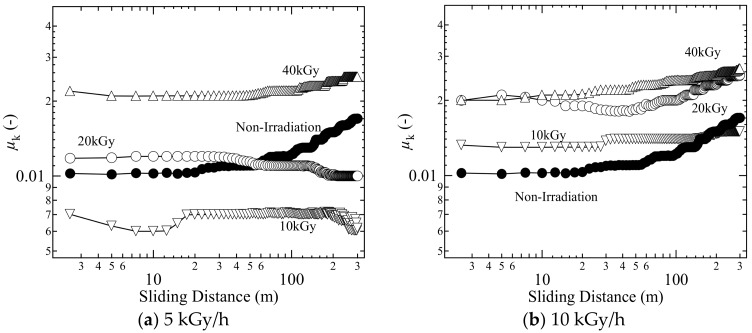
Coefficient of friction of gamma ray irradiated gels with (**a**) 5 kGy/h and (**b**) 10 kGy/h in reciprocating friction tests at room temperature; close circle, non-irradiation gel; inverted triangle, irradiance = 10 kGy; open circle, irradiance = 20 kGy; triangle, irradiance = 40 kGy.

To investigate the effect of gamma ray irradiation on frictional properties, the reciprocating test at room temperature was conducted first. The changes in coefficient of friction, *µ*_k_, are shown in Figure 2. The samples as shown in Figure 2a,b were prepared at the dose rate of 5 and 10 kGy/h, respectively. At the dose rate of 5 kGy/h, *µ*_k_ for 10 kGy irradiance was noticed to be reduced from non-irradiation. To compare friction levels among irradiated gels, *µ*_k_ increased as the irradiance increased at each dose rate. In addition, it is also noticed that the increase in *µ*_k_ depended on the dose rate. At the same irradiance, the gel at a high dose rate showed a high *µ*_k_ except at 40 kGy.

The changes in *µ*_k_ with irradiation are considered to be related to the variation in shearing resistance of the irradiated gels. As the surface solidifies due to the increase in chemical cross-linking points, the shear strength/resistance increases, which increases the *µ*_k_. However, the reduction in friction for 10 kGy at 5 kGy/h from non-irradiation level appeared to be related to the improvement of shearing strength to frictional force. The high friction at high dose rate suggests that the surface physical properties intensely changed by receiving a lot of gamma rays in a short time. The size and distribution of cross-linking points on the surface changed, resulting from the variation in the amount of oxygen around the gel at the time of irradiation with gamma rays. Therefore, in order to maintain the friction characteristics of PVA CD on FT hybrid gels, it is important to reduce the dose rate and irradiance.

#### 2.2.2. Reciprocating Test at 37 °C

**Figure 3:**
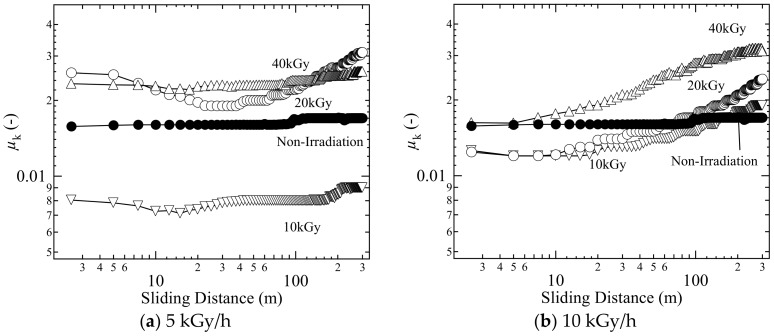
Coefficient of friction of gamma ray irradiated gels with (**a**) 5 kGy/h and (**b**) 10 kGy/h in reciprocating friction tests at body temperature (about 37 °C); close circle, non-irradiation gel; inverted triangle, irradiance = 10 kGy; open circle, irradiance = 20 kGy; triangle, irradiance = 40 kGy.

To investigate the frictional property of gamma ray irradiation gels in an environment close to human body, the temperature of the lubricating liquid was raised at 37 °C. The results are shown in Figure 3. 

The samples as shown in Figure 3a,b were prepared at dose rate 5 and 10 kGy/h, respectively. Compared with the result at room temperature (Figure 2), the non-irradiation gel showed a higher *µ*_k_ at 37 °C. On the other hand, the gamma ray irradiated gel at 37 °C did not show evident changes in *µ*_k_ from that at room temperature, except for 20 kGy.

The deterioration of frictional properties of the physically cross-linked gels at 37 °C is possibly caused by a decrease in shear strength and an increase in adhesion, because the gel slightly dissolves due to the breakage of physical cross-linking near body temperature. In the gels irradiated with gamma rays, the physical cross-linking destruction was suppressed by the formation of chemically cross-linking points. The above results revealed that the gels irradiated using gamma rays of 10 kGy at 5 kGy/h exhibited better friction characteristics than the conventional (non-irradiated) CD on FT hybrid gels in the reciprocating frictional test.

### 2.3. Wear Properties

**Figure 4:**
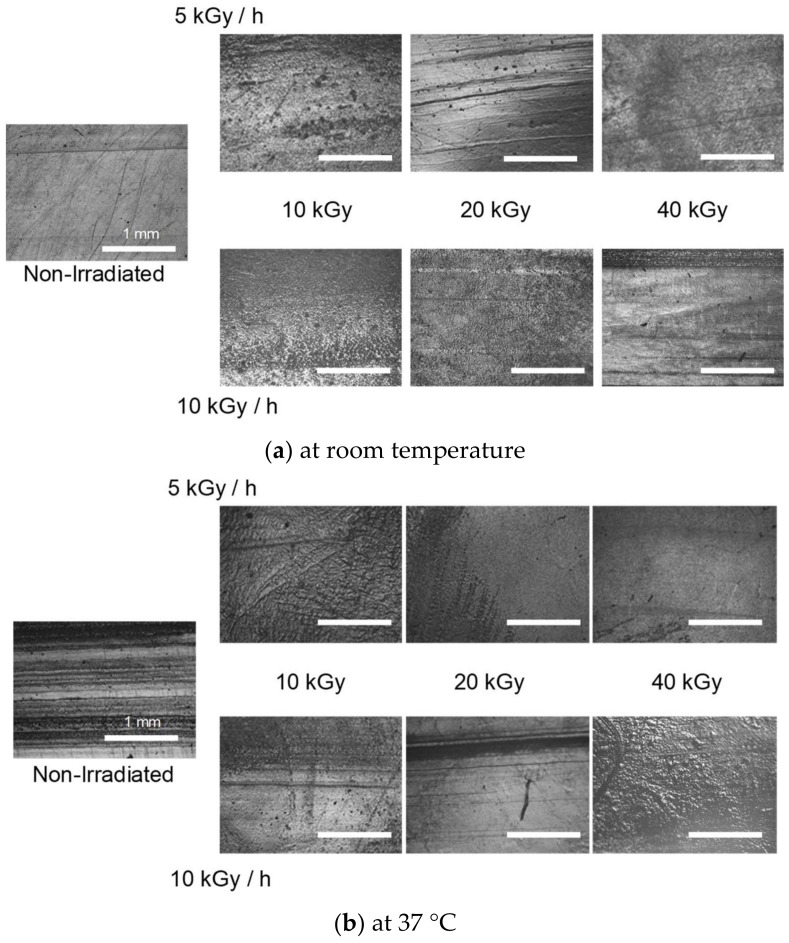
The photomicrograph of the surface of the gel after reciprocating friction tests at (**a**) room temperature and (**b**) body temperature. Scale bar is 1 mm.

As far as application as artificial cartilage is concerned, wear is as important as friction. Wear should be kept to the minimum in order to suppress adverse effects in the body. Therefore, the sample surface after the reciprocating friction test was observed using a phase contrast microscope. Figure 4 shows a phase contrast micrograph of wear traces. Figure 4a is a photomicrograph of the gel surface after the reciprocating friction test at room temperature (Figure 2). On the surface of gels irradiated at 10 kGy, little scratches are detected. By increasing gamma ray irradiance, scratches caused by wear increased after the reciprocating friction test.

Figure 4b is a photomicrograph of the gel surface after the reciprocating friction test at body temperature (Figure 3). The distinct scratches were formed on the surface of the non-irradiated gel surface at 37 °C. In contrast, the irradiated surface features revealed that wear was suppressed by gamma ray irradiation. 

The scratches on the irradiated gel surface except for 10 kGy after rubbing at room temperature are regarded to be caused by the excessive increase in the surface hardness via the formation of chemical cross-linking points. Wear suppression at 37 °C by gamma ray irradiation is considered to be actualized by the improvement in gel structure. The chemical cross-linking point was not destroyed at 37 °C, and the decrease in the shear strength of the surface was suppressed.

### 2.4. Discussion on Friction and Wear Properties

As discussed above, low dose rate and low irradiance appear to actualize good friction and wear characteristics for clinical applications at body temperature.

For non-irradiated gel at 37 °C, the increase in friction and surface scratching is considered to have been brought about by a decrease in shear strength and an increase in adhesive force, as a result of the local breakage of physical cross-linking accompanied with some dissolution of gel. In order to maintain low friction and minimal wear at 37 °C, appropriate improvement of gel structure and properties is required but excessive hardening with cross-linking may deteriorate the tribological properties of PVA CD on FT hybrid gel. The irradiation treatment by gamma ray at 10 kGy and 5 kGy/h is expected to be an optimum condition for gel structure, although further investigation is required to elucidate the detailed improving mechanism with gamma irradiation.

## 3. Conclusions

Because the chemically cross-linking point was introduced by gamma ray irradiation, the water content decreased, which was still maintained about 70%. 

Gamma ray irradiated gels at appropriate conditions showed superior friction and wear characteristics near body temperature. Gels at low dose rate were excellent in terms of their friction and wear characteristics. As the irradiance increased, the friction coefficient increased.

It is concluded that sterilization treatment with low dose rate and irradiance is suitable for applying PVA gels as candidate materials for artificial cartilage. However, it is necessary to confirm that the sterilization is completed under this condition.

## 4. Materials and Methods 

### 4.1. Sample Preparation

#### 4.1.1. PVA CD on FT Hybrid Gel

PVA pre-gel solution (15 wt %) was prepared by dissolving PVA powder (PVA117; Kuraray Co., Ltd., Tokyo, Japan, degree of polymerization: 1700, degree of hydrolysis: 98 to 99 mol %) in pure water at a temperature around 90 °C over a period of 2 h. The PVA pre-gel solution (15.0 g) was then decanted into a polystyrene dish (with an inner diameter of 85 mm). The pre-gel solution was frozen at −20 °C for 8 h and then thawed at 4 °C for 9 h. This process was repeated 4 times. After the freezing and thawing process, the second CD gel layer was prepared. The PVA pre-gel solution (15.0 g) was decanted on the FT gel layer and dried at 8 °C and 50%RH for 7 days in a temperature and humidity controlled chamber (SU-242, ESPEC, Osaka, Japan), before it was treated at 20 °C and 40%RH until the weight became almost constant. The obtained CD on FT hybrid PVA hydrogel film was soaked in 1 L pure water for 48 h.

#### 4.1.2. Gamma Ray-Irradiation

After swelling the fabricated gels in pure water over 24 h, the samples in water were irradiated by gamma rays from a 60Co radiation source. The irradiation experiments were carried out at National Institutes for Quantum and Radiological Science and Technology (Watanuki 1233, Takasaki, Gunma, Japan). Total dose irradiances were 10, 20, and 40 kGy. The dose rates were about 5 and 10 kGy/h.

### 4.2. Water Contents

To analyze the swelling property, square samples (10 mm × 10 mm) were cut out from the swollen gels. The weight of the swollen sample, *W*_t_, was measured. After the measurement, the gels were dried at 60 °C for 24 h in a temperature-controlled chamber, and the weight of dried sample, *W*_d_, was measured. From these measurements, water content was calculated as 1 − (*W*_d_/*W*_t_).

### 4.3. Reciprocating Friction Test

For each swollen gel, a ball-on-plate reciprocating friction test was conducted by using a friction tester (TriboGear TYPE:38, HEIDON). Rectangle samples (40 mm × 10 mm) were cut out from the swollen gel. The lubricant temperatures were room temperature and 37 °C. Sliding speed was 20 mm/s. Total sliding distance was 300 m (stroke: 25 mm; total cycle: 6000). Vertical load was 5.88 N. The lubricant was pure water. These conditions were selected according to a previous study [13] to evaluate friction and wear properties of PVA hydrogels in mixed or boundary lubrication mode. Polycrystalline alumina ceramic ball of 26 mm diameter (surface roughness *R*_a_ < 0.01 μm) was used as an upper ball specimen. From the measured tangential force, coefficient of friction, *µ*_k_, was calculated.
